# The Effect of HIV on the Association of Hyperglycaemia and Active Tuberculosis in Zambia, a Case–Control Study

**DOI:** 10.1007/s44197-024-00236-2

**Published:** 2024-05-07

**Authors:** Sarah Lou Bailey, Sian Floyd, Maina Cheeba-Lengwe, Kwitaka Maluzi, Kasanda Chiwele-Kangololo, Deborah Kaluba-Milimo, Modupe Amofa-Sekyi, John S. Yudkin, Peter Godfrey-Faussett, Helen Ayles

**Affiliations:** 1 LSHTM TB Centre and Department of Clinical Research, London School of Hygiene and Tropical Medicine, London, UK; 2 Zambart, Lusaka, Zambia; 3 Division of Medicine, University College London, London, UK

**Keywords:** Diabetes mellitus, Hyperglycaemia, Tuberculosis, HIV, Africa, Epidemiology

## Abstract

**Objectives:**

To determine if HIV modifies the association between hyperglycaemia and active tuberculosis in Lusaka, Zambia.

**Methods:**

A case–control study among newly—diagnosed adult tuberculosis cases and population controls in three areas of Lusaka. Hyperglycaemia is determined by random blood glucose (RBG) concentration measured at the time of recruitment; active tuberculosis disease by clinical diagnosis, and HIV status by serological result. Multivariable logistic regression is used to explore the primary association and effect modification by HIV.

**Results:**

The prevalence of RBG concentration ≥ 11.1 mmol/L among 3843 tuberculosis cases was 1.4% and among 6977 controls was 1.5%. Overall, the adjusted odds ratio of active tuberculosis was 1.60 (95% CI 0.91–2.82) comparing those with RBG concentration ≥ 11.1– < 11.1 mmol/L. The corresponding adjusted odds ratio among those with and without HIV was 5.47 (95% CI 1.29–23.21) and 1.17 (95% CI 0.61–2.27) respectively; *p*-value for effect modification by HIV = 0.042. On subgroup analysis, the adjusted odds ratio of smear/Xpert-positive tuberculosis was 2.97 (95% CI 1.49–5.90) comparing RBG concentration ≥ 11.1– < 11.1 mmol/L.

**Conclusions:**

Overall, no evidence of association between hyperglycaemia and active tuberculosis was found, though among those with HIV and/or smear/Xpert-positive tuberculosis there was evidence of association. Differentiation of hyperglycaemia caused by diabetes mellitus and stress-induced hyperglycaemia secondary to tuberculosis infection is important for a better understanding of these findings.

## Introduction

HIV and hyperglycaemia are independently associated with an increased risk of active tuberculosis (TB) [1, 2]. However, the dual effect of HIV and hyperglycaemia on the risk of developing TB disease is unclear. A systematic review [3] identified two prior studies investigating the effect of HIV on the association between hyperglycaemia or diabetes mellitus (DM) and tuberculosis [4, 5]. Another systematic review [6] identified a further two studies that stratified their estimates by HIV status [7, 8], and we identified one additional study that has investigated these associations [9]. The findings differed for each study depending on the analysis strategy and the method used to measure glycaemia.

Global guidelines exist for the care and control of co-existing diabetes mellitus and tuberculosis, but these were developed using evidence from studies largely based in low HIV prevalence settings [2, 10, 11]. If HIV does modify the association, this could lead to a different overall association in areas of high HIV prevalence. Understanding this could allow location-specific guidelines to be developed and implemented, to optimise the care and control of DM and TB.

This study aims to determine whether HIV modifies the association between hyperglycaemia and active TB in Lusaka, Zambia.

## Methods

### Study Design and Setting

This unmatched case–control study took place among adults in three urban communities in Lusaka, Zambia. Each community has a high incidence of TB and a high prevalence of HIV. The exposure of interest was hyperglycaemia and the outcome was diagnosed active TB disease.

### Cases

Consecutive newly—diagnosed TB cases who had not yet started TB treatment or were within 2 days of starting TB treatment were recruited from National TB Programme government clinics between September 2013 and September 2015. A TB case was defined as any person presenting to a TB clinic with a clinical diagnosis of TB (pulmonary or extra-pulmonary) with or without microbiological confirmation of TB and prescribed a full course of antitubercular chemotherapy [12]. This is the definition used in the study clinics—use of this aimed to facilitate translation of results to the clinical setting. At the time of data collection the study clinics were using sputum smear, Xpert MTB/RIF or both to diagnose TB, depending on the availability of reagents and equipment. Exclusion criteria for cases were age < 18 years, commenced TB treatment > 2 days prior to recruitment or inability to give consent due to disability/incapacitation. All eligible cases were identified in the TB clinic and invited to participate during a routine clinic attendance.

### Controls

Unmatched controls were recruited between January and December 2010 as part of a cross-sectional survey that measured prevalent TB for a large cluster randomised trial (the ZAMSTAR trial) [13, 14]. They were sampled randomly from all adults living in the catchment areas of the clinics, using a 2-stage cluster sampling technique within each community. Exclusion criteria for controls were age < 18 years, refusal to submit a respiratory sample, diagnosed TB at the time of the survey (culture positive sputum for *Mycobacterium tuberculosis*), currently on treatment for TB at the time of the survey (self-report), inability to give consent due to disability/incapacitation or any persons living in institutional settings. All eligible controls were recruited in their homes.

### Data Collection

Glycaemia was determined by random capillary blood glucose (RBG) sampling measured at the time of recruitment. An Optium Xceed point-of-care glucometer was used for controls and an Accu-Chek Aviva point-of-care glucometer was used for cases. Manufacture of the former had been discontinued between the start of data collection for controls and cases so the Aviva was identified as being a similar and well-performing option instead. The time of last oral intake (excluding water) was recorded. RBG concentration was measured during daylight hours and throughout the year. Research staff were trained on the use of the glucometers and were required to undergo proficiency testing. Standardised control solution was used for performance checks on test strips and meters. The validity of glucose measurement was assessed for the Aviva glucometer, giving an intraclass correlation of 0.996 (95% CI 0.991–0.999) for intra-operator variability and 0.983 (95% CI 0.954–0.995) for inter-operator variability. All research assistants contributed to this assessment, using known normoglycaemic volunteers and a standard hyperglycaemic specimen. For intra-operator variability each research assistant repeated the test 5 consecutive times on 13 subjects/specimens. For inter-operator variability research assistants each performed the test on a single subject/specimen in the same place at the same time for 7 subjects/specimens.

For a stratified subset of cases, fasting blood glucose (FBG) concentration was measured within 3 days of commencement of TB treatment and again 3 months later, to indicate if the hyperglycaemia was transient or persistent.

HIV was measured using point-of-care rapid blood-based kits. Determine™ HIV-1/2 was used as the first line test and if positive Uni-Gold™ HIV was used as a confirmatory test. Self-reported use of antiretroviral therapy (ART) was recorded for individuals who were already known to be infected with HIV.

Participants who tested positive for HIV or were identified to have abnormal blood glucose were referred to local healthcare facilities for management.

In order to adjust for potential confounding factors, a structured questionnaire was used to obtain information on age, sex, smoking history, household socio-economic position and education level. Height, weight and waist circumference were measured using standardised methods.

Data were electronically entered directly onto personal digital assistants by research assistants at the time of data collection, using pre-programmed questionnaires and result sheets with error and range checks. All data were downloaded into a SQL (structured query language) database and exported into Stata.

All questions, measurement tools (HIV testing kits but not glucometers) and test standard operating procedures were identical for cases and controls.

### Ethics

Each participant was required to give written informed consent. Ethics approval was granted from the London School of Hygiene and Tropical Medicine Ethics Committee and the University of Zambia Biomedical Research Ethics Committee.

### Statistical Analysis

The study sample size was calculated to give sufficient power for determination of effect modification by HIV. Principal components analysis was used to create a measure of household socio-economic position. Hyperglycaemia was initially examined with RBG concentration as an ordered categorical variable. We then used the RBG cut-off 11.1 mmol/L to explore hyperglycaemia as a binary variable.

Unadjusted and adjusted odds ratios of the association between hyperglycaemia and active TB were estimated using logistic regression analysis. Interaction terms were used to assess for effect modification by HIV, adjusting for variables considered a priori as potential confounding factors.

Restriction of the outcome definition separately to sputum smear-positive or Xpert MTB/RIF positive tuberculosis, smear-negative and Xpert MTB/RIF negative pulmonary TB, and extrapulmonary TB were identified a priori for sub-group analyses, as was exploration of the impact of ART use on HIV as an effect modifier.

Potential bias was assessed using chi-squared tests of association of glycaemia with time of last oral intake before measurement and time of day of measurement of RBG concentration, each comparing cases to controls. Intra- and inter-operator variability were assessed for absolute agreement using a one-way and a two-way random-effects model respectively [15].

## Results

**Fig. 1:**
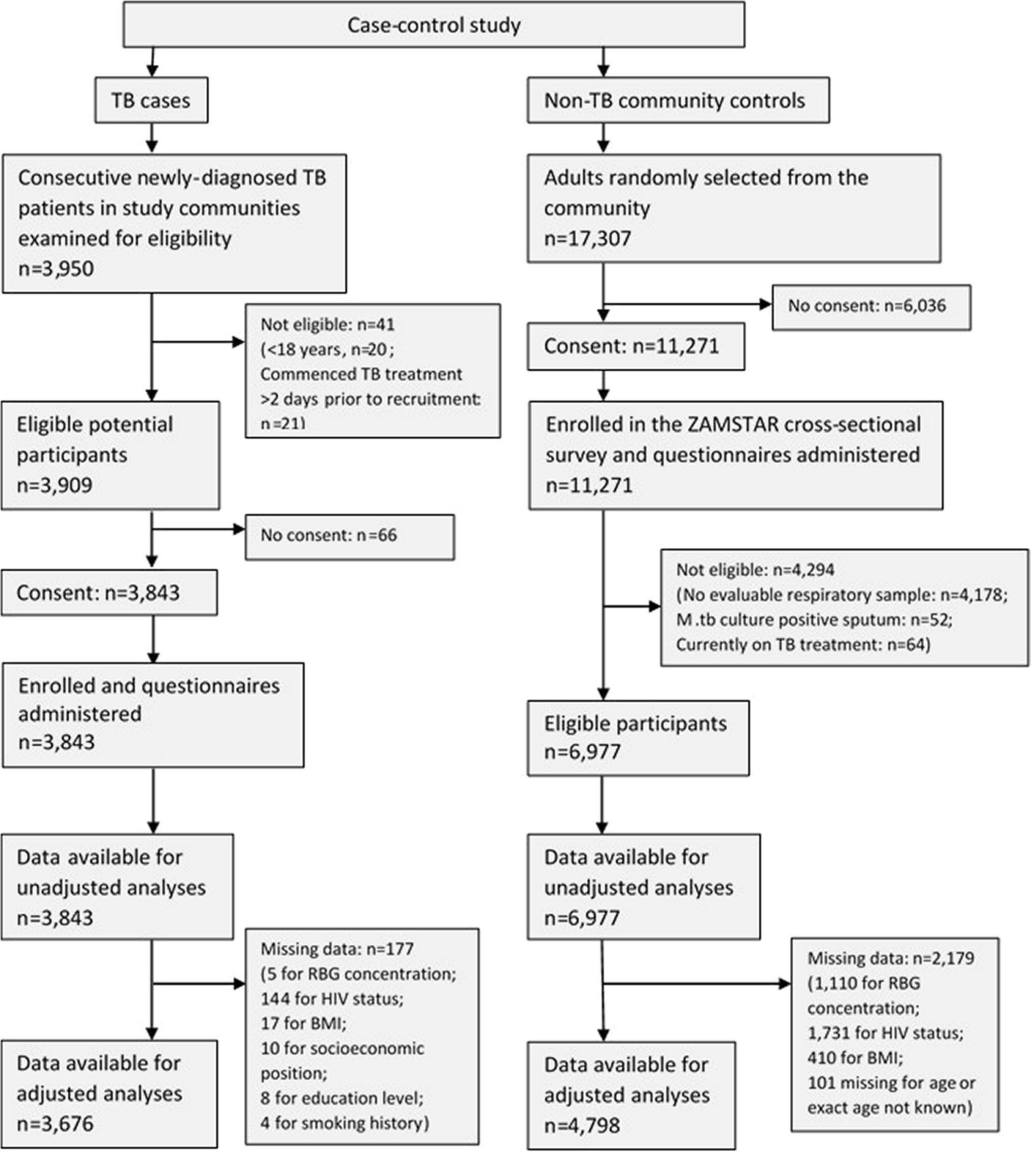
Number and flow of cases and controls

There were 3909 eligible TB cases identified, of whom 3843 (98.3%) consented to participate. There were 11,271 adults randomly selected from the three study communities and enrolled in the ZAMSTAR survey, of whom 6977 (61.9%) were eligible to be control participants for this study (Fig. 1).

The characteristics of participants are shown in Table 1. Among cases and controls 1.4% and 1.5% respectively had a RBG concentration ≥ 11.1 mmol/L (Table 1). 65% of cases and 18% of controls were living with HIV.

**Table 1:** Logistic regression estimates of the unadjusted and adjusted odds ratios of tuberculosis, stratified by HIV status

Characteristic		Cases n (%)	Controls n (%)	Unadjusted OR (95% CI)	*p*-value	Adjusted OR (95% CI)	*p*-value
Overall		3843 (100)	6977 (100)	–	–	–	–
Glucose concentration (mmol/L)*	< 5.6	2255 (58.8)	3069 (52.3)	1	< 0.001 TFT: < 0.001 DFT: < 0.001	1	< 0.001 TFT: 0.051 DFT: < 0.001
	5.6–6.9	1089 (28.4)	1845 (31.5)	0.80 (0.73–0.88)		0.86 (0.74–1.00)	
	7.0–8.9	352 (9.2)	755 (12.9)	0.63 (0.55–0.73)		0.62 (0.50–0.77)	
	9.0–11.0	89 (2.3)	109 (1.9)	1.11 (0.84–1.48)		1.36 (0.84–2.21)	
	> 11.0	53 (1.4)	89 (1.5)	0.81 (0.57–1.14)		1.46 (0.83–2.58)	
HIV status*	Uninfected	1287 (34.8)	4320 (82.4)	1	< 0.001	1	< 0.001
	Infected	2412 (65.2)	926 (17.7)	8.74 (7.93–9.64)		9.35 (8.07–10.85)	
HIV uninfected	< 5.6	803 (62.4)	2258 (53.6)	1	–	1	–
	5.6–6.9	330 (25.7)	1297 (30.8)	0.72 (0.62–0.83)	< 0.001	0.86 (0.71–1.04)	0.125
	7.0–8.9	106 (8.2)	520 (12.3)	0.57 (0.46–0.72)	< 0.001	0.63 (0.47–0.85)	0.002
	9.0–11.0	24 (1.9)	71 (1.7)	0.95 (0.59–1.52)	0.832	1.33 (0.71–2.49)	0.372
	> 11.0	23 (1.8)	71 (1.7)	0.91 (0.57–1.47)	0.701	1.08 (0.56–2.09)	0.826
HIV infected	< 5.6	1365 (56.6)	447 (49.2)	1	–	1	–
	5.6–6.9	727 (30.2)	316 (34.8)	0.75 (0.64–0.89)	0.001	0.86 (0.68–1.09)	0.209
	7.0–8.9	232 (9.6)	125 (13.8)	0.61 (0.48–0.77)	< 0.001	0.61 (0.43–0.85)	0.004
	9.0–11.0	60 (2.5)	16 (1.8)	1.23 (0.70–2.15)	0.474	1.41 (0.66–3.02)	0.371
	> 11.0	27 (1.1)	5 (0.6)	1.77 (0.68–4.62)	0.245	4.94 (1.16–21.02)	0.031
Glucose < 5.6 mmol/L	HIV uninfected	803 (37.0)	2258 (83.5)	1	< 0.001	1	< 0.001
	HIV infected	1365 (63.0)	447 (16.5)	8.59 (7.51–9.82)		9.26 (7.67–11.19)	
Glucose 5.6–6.9 mmol/L	HIV uninfected	330 (31.2)	1297 (80.4)	1	< 0.001	1	< 0.001
	HIV infected	727 (68.8)	316 (19.6)	9.04 (7.56–10.81)		9.26 (7.18–11.95)	
Glucose 7.0–8.9 mmol/L	HIV uninfected	106 (31.4)	520 (80.6)	1	< 0.001	1	< 0.001
	HIV infected	232 (68.6)	125 (19.4)	9.10 (6.73–12.31)		8.86 (5.85–13.44)	
Glucose 9.0–11.0 mmol/L	HIV uninfected	24 (28.6)	71 (81.6)	1	< 0.001	1	< 0.001
	HIV infected	60 (71.4)	16 (18.4)	11.09 (5.40–22.79)		9.84 (3.73–25.93)	
Glucose > 11.0 mmol/L	HIV uninfected	23 (46.0)	71 (93.4)	1	< 0.001	1	< 0.001
	HIV infected	27 (54.0)	5 (6.6)	16.67 (5.75–48.30)		42.48 (8.74–206.50)	
Age (years)	18–24	555 (14.4)	2771 (40.3)	1	< 0.001	1	< 0.001
	25–29	751 (19.5)	1222 (17.8)	3.07 (2.70–3.49)		2.68 (2.20–3.26)	
	30–34	861 (22.4)	813 (11.8)	5.29 (4.63–6.04)		3.46 (2.80–4.28)	
	35–39	755 (19.7)	573 (8.3)	6.58 (5.71–7.58)		4.52 (3.59–5.68)	
	40–49	612 (15.9)	727 (10.6)	4.20 (3.65–4.84)		2.96 (2.34–3.74)	
	50 +	309 (8.0)	770 (11.2)	2.00 (1.71–2.35)		2.13 (1.66–2.74)	
Sex	Male	2605 (67.8)	1955 (28.0)	1	< 0.001	1	< 0.001
	Female	1238 (32.2)	5022 (72.0)	0.19 (0.17–0.20)		0.19 (0.16–0.22)	
Highest level of education	None	387 (10.1)	446 (6.4)	1	< 0.001	1	< 0.001
	Grade 1–7	1388 (36.2)	2474 (35.5)	0.65 (0.56–0.75)		0.42 (0.32–0.56)	
	Grade 8–12	1969 (51.3)	3645 (52.2)	0.62 (0.54–0.72)		0.31 (0.24–0.41)	
	College/University	91 (2.4)	412 (5.9)	0.25 (0.2–0.33)		0.10 (0.07–0.16)	
Smoking history	Never smoked	2863 (74.6)	6188 (88.7)	1	< 0.001	1	0.326
	Current or ex-smoker	976 (25.4)	789 (11.3)	2.67 (2.41–2.97)		1.10 (0.91–1.32)	
Body mass index [weight (kg)/height^2^ (m)]	Healthy weight (18.5–24.9)	1985 (51.9)	4106 (62.5)	1	< 0.001	1	< 0.001
	Underweight (< 18.5)	1659 (43.4)	541 (8.2)	6.34 (5.68–7.09)		6.49 (5.49–7.68)	
	Overweight (25–29.9)	129 (3.4)	1265 (19.3)	0.21 (0.17–0.25)		0.24 (0.19–0.31)	
	Obese (≥ 30)	53 (1.4)	655 (10.0)	0.17 (0.13–0.22)		0.23 (0.16–0.34)	

*OR* odds ratio, *CI* confidence interval, *TFT* test for linear trend, *DFT* test for departure from linear trend

*Estimates from model with no interaction. All other estimates taken from model with HIV interaction; Adjusted analyses adjusted for all variables shown, plus household socioeconomic position and community; 167 cases and 2179 controls excluded from adjusted analyses due to missing data; *p*-value for interaction, unadjusted analysis = 0.707; *p*-value for interaction, adjusted analysis = 0.390

Comparing participants with RBG ≥ 11.1 mmol/L to those with RBG < 11.1 mmol/L, the unadjusted odds ratio of TB was 0.91 (95% CI 0.65–1.28). When analysed with RBG concentration as an ordered categorical variable, there was evidence of an association between hyperglycaemia and tuberculosis, but there was strong evidence that the association did not follow a linear trend. We therefore did not examine the association with RBG concentration as a continuous variable. On unadjusted analysis there was no evidence to suggest that the odds of TB for the effect of hyperglycaemia differed between individuals infected and uninfected with HIV (Table 1).

The adjusted odds of TB was 1.60 times higher in those with RBG ≥ 11.1 mmol/L compared to those with RBG < 11.1 mmol/L (95% CI 0.91–2.82), adjusting for the effect of age, gender, education, socioeconomic position, body mass index, smoking history and community. As a categorical variable there remained strong evidence of a non-linear association. Among individuals with HIV, the adjusted odds of TB was 5.47 times higher in those with RBG ≥ 11.1 mmol/L compared to those with RBG < 11.1 mmol/L (95% CI 1.29–23.21). Among individuals without HIV, the adjusted odds of TB was 1.17 times higher for the same comparison (95% CI 0.61–2.27). The *p*-value for interaction was 0.042. The main confounding factors were age, sex and body mass index.

When the analysis was restricted to separate TB categories, there was evidence of association between hyperglycaemia and sputum smear/Xpert-positive tuberculosis, and this association was stronger in individuals infected than uninfected with HIV (*p*-value for interaction = 0.028, Table 2).

**Table 2:** Logistic regression estimates of the unadjusted and adjusted odds ratios of smear/Xpert-positive, smear/Xpert-negative and extrapulmonary tuberculosis, stratified by HIV status

Characteristic		Cases n (%)	Controls n (%)	Unadjusted OR (95% CI)	*p*-value	Adjusted OR (95% CI)	*p*-value
Sputum smear/Xpert-positive tuberculosis							
RBG concentration (mmol/L)*	< 11.1	1533 (98.5)	5778 (98.5)	1	0.911	1	0.003
	≥ 11.1	23 (1.5)	89 (1.5)	0.97 (0.61–1.55)		2.97 (1.49–5.90)	
HIV uninfected	RBG < 11.1	588 (98.3)	4146 (98.3)	1	0.984	1	0.153
	RBG ≥ 11.1	10 (1.7)	71 (1.7)	0.99 (0.51–1.94)		1.87 (0.79–4.39)	
HIV infected	RBG < 11.1	879 (98.8)	904 (99.5)	1	0.132	1	0.001
	RBG ≥ 11.1	11 (1.2)	5 (0.6)	2.26 (0.78–6.54)		12.43 (2.63–58.69)	
Sputum smear/Xpert-negative tuberculosis							
RBG concentration (mmol/L)*	< 11.1	1903 (98.7)	5778 (98.5)	1	0.593	1	0.518
	≥ 11.1	26 (1.4)	89 (1.5)	0.89 (0.57–1.38)		1.27 (0.61–2.63)	
HIV uninfected	RBG < 11.1	592 (98.2)	4146 (98.3)	1	0.803	1	0.949
	RBG ≥ 11.1	11 (1.8)	71 (1.7)	1.09 (0.57–2.06)		0.97 (0.42–2.27)	
HIV infected	RBG < 11.1	1260 (98.9)	904 (99.5)	1	0.182	1	0.153
	RBG ≥ 11.1	14 (1.1)	5 (0.6)	2.01 (0.72–5.60)		3.55 (0.62–20.19)	
Extrapulmonary tuberculosis							
RBG concentration (mmol/L)*	< 11.1	320 (98.8)	5778 (98.5)	1	0.675	1	0.324
	≥ 11.1	4 (1.2)	89 (1.5)	0.81 (0.30–2.22)		1.88 (0.58–6.13)	
HIV uninfected	RBG < 11.1	73 (97.3)	4146 (98.3)	1	0.518	1	0.573
	RBG ≥ 11.1	2 (2.7)	71 (1.7)	1.60 (0.39–6.65)		1.54 (0.34–6.92)	
HIV infected	RBG < 11.1	226 (99.1)	904 (99.5)	1	0.576	1	0.321
	RBG ≥ 11.1	2 (0.9)	5 (0.6)	1.60 (0.31–8.30)		2.92 (0.35–24.26)	

*OR* odds ratio, *CI* confidence interval

*Estimates from model with no interaction; *p*-values for interaction: smear/Xpert-positive TB = 0.028, smear/Xpert-negative TB = 0.172, extrapulmonary TB = 0.628

Among individuals infected with HIV, 36.7% of cases and 21.2% of controls were taking ART. There was evidence of association between hyperglycaemia and active tuberculosis among individuals who were HIV-positive and not currently taking ART, but not among individuals who were HIV-positive and taking ART (Table 3).

**Table 3:** Logistic regression estimates of the unadjusted and adjusted odds ratios of tuberculosis, stratified by HIV status and use of antiretroviral therapy

Characteristic		Cases n (%)	Controls n (%)	Unadjusted OR (95% CI)	*p*-value	Adjusted OR (95% CI)	*p*-value
RBG concentration (mmol/L)*	< 11.1	3785 (98.6)	5778 (98.5)	1	0.584	1	0.104
	≥ 11.1	53 (1.4)	89 (1.5)	0.91 (0.65–1.28)		1.60 (0.91–2.82)	
HIV uninfected	RBG < 11.1	1263 (98.2)	4146 (98.3)	1	0.800	1	0.574
	RBG ≥ 11.1	23 (1.8)	71 (1.7)	1.06 (0.66–1.71)		1.21 (0.62–2.34)	
HIV infected and currently taking ART	RBG < 11.1	878 (99.3)	193 (98.5)	1	0.248	1	0.885
	RBG ≥ 11.1	6 (0.7)	3 (1.5)	0.44 (0.11–1.77)		0.85 (0.09–7.79)	
HIV infected and not currently taking ART	RBG < 11.1	1506 (98.6)	711 (99.7)	1	0.031	1	0.013
	RBG ≥ 11.1	21 (1.4)	2 (0.3)	4.96 (1.16–21.2)		16.79 (1.8–156.75)	

*OR* odds ratio, *CI* confidence interval, *ART* antiretroviral therapy

*Estimates from model with no interaction

FBG concentration was measured in 232 participants with TB at baseline and 3 months later. Nine participants had hyperglycaemia at baseline (FBG ≥ 7.0 mmol/L) and 4 (44.4%) of these had persistent hyperglycaemia 3 months later. Three (75%) participants with persistent hyperglycaemia were uninfected with HIV. Four (80%) participants with transient hyperglycaemia were infected with HIV.

Participants reported the time of last oral intake prior to measurement of RBG concentration to be a mean of 4.9 h (standard deviation 4.6 h). There was no evidence of a difference in time (< or ≥ 6 h) of last oral intake between participants with RBG < and ≥ 11.1 mmol/L (*p* = 0.815), nor between individuals with and without TB (*p* = 0.793). There was no evidence of a difference in the time (hour) of measurement between participants with RBG < and ≥ 11.1 mmol/L (*p* = 0.832).

## Discussion

In this case–control study in Lusaka, Zambia, we found no evidence of association between hyperglycaemia and active tuberculosis, except for when TB was restricted to individuals with smear/Xpert-positive pulmonary TB. There was evidence of effect modification by HIV for the association between hyperglycaemia and active TB. When adjusted for confounding factors, the association was stronger among individuals infected with HIV than among uninfected individuals.

When analysed as an ordered categorical variable with pre-defined categories, there was evidence of a non-linear association between hyperglycaemia and tuberculosis in our study population, as individuals with RBG concentration 7.0–8.9 mmol/L had a lower odds of TB than individuals with lower or higher RBG concentration (*p* < 0.001). This was an unexpected finding and may be due to chance as there is no biological reason to explain this pattern.

Although our primary association findings are not in keeping with the findings of the most recent systematic review, which reported a pooled odds ratio of 2.77 for the association between hyperglycaemia and tuberculosis in Africa [6], they do mirror the findings of some studies in nearby communities in Guinea-Bissau [16], South Africa [9] and Tanzania [5]. Boillat-Blanco et al. in Tanzania found a positive association between hyperglycaemia and tuberculosis at the time of TB treatment initiation, but the association disappeared when measurement of diabetes was repeated 5 months after TB treatment initiation, suggesting that the initial positive association was due to an increase in stress-induced hyperglycaemia among TB cases secondary to acute TB infection rather than due to DM [5].

Our findings of a stronger association among HIV infected than uninfected individuals are in keeping with Oni’s findings [9] and could suggest that HIV and hyperglycaemia work synergistically to increase an individual’s risk of TB. Another possible explanation is an increase in stress-induced hyperglycaemia among newly—diagnosed TB cases, as seen in Boillat-Blanco’s study [5]. It is plausible that the most unwell newly diagnosed TB cases, and therefore the most likely to have stress-induced hyperglycaemia, could be found among individuals with HIV and smear/Xpert-positive pulmonary TB.

This study used a single RBG concentration to measure hyperglycaemia. This method is simple, quick, and minimises participant inconvenience, so is ideal for use in large community-based studies. However, use of this method is also a limitation of the study as it is not as sensitive for diabetes diagnosis as other glycaemia measures. We chose to go ahead with using this method because all other methods would have been challenging to perform on a large scale in the community and could have led to selection bias if considered to be unacceptable to healthy control participants. An alternative could have been the use of a clinic-based control population as a proxy to community controls, but this too could have led to selection bias. Assessing shifts in proportions of glucose concentration between cases and controls in addition to using a binary cut-off definition of hyperglycaemia has limited potential misclassification error that could exist with the use of this less sensitive measurement.

Our assessment of intra- and inter-operator variability suggests that measurement of point-of-care RBG among cases in this study was consistent and valid. We explored the possibility of also undertaking laboratory validation of glucose measurements but this was not possible in our setting, as point-of-care glucose measurement is the principal method for measuring glycaemia in the community and centrally. The laboratory alternatives were therefore not equipped to offer a reliable benchmark. This lack of laboratory validation of glucose measurements is a limitation of our study. Finding a solution to this challenge in our setting would be valuable for future similar studies.

The temporal space between recruitment of controls and cases is another potential source of bias and limitation of our study, though the study communities have relatively stable populations and to our knowledge there were no major changes in the prevalence of hyperglycaemia, diabetes or HIV, or the incidence of TB during the study period. Any potential change in use of ART is unlikely to have had a major impact on hyperglycaemia, as protease inhibitors—which are associated with the development of glucose disorders [17]—are not included among first-line ART regimes in Zambia [18]. This is supported by exploration of the impact of ART on the associations studied. Any bias introduced by the recruitment gap is therefore likely to have been, at the most, minimal. However, an unforeseen consequence of the gap was the discontinuation of the initial glucometer model used for control participants. It was therefore necessary to use a different model for case participants. We chose a similar model to minimise any potential variability between models and so any difference in measurement of glycaemia is likely to be small rather than large.

Complete data were unavailable for analysis for a substantial proportion of control participants, reasons for which are discussed elsewhere [8, 19, 20]. This has resulted in reduced study power but is unlikely to have introduced bias to our study results.

Control participants are representative of the general population and case participants are representative of TB cases in each community. The findings from this study are therefore generalizable to the study communities and likely also to communities elsewhere in sub-Saharan Africa with similar high incidence of TB and high prevalence of HIV.

## Conclusions

Overall, no evidence of association between hyperglycaemia and active tuberculosis was found in this study population, though among those with HIV and those with smear/Xpert-positive pulmonary TB there was evidence of an association. Differentiation of hyperglycaemia caused by diabetes mellitus and stress-induced hyperglycaemia secondary to tuberculosis infection is important to understand these data further. Future similar studies should aim to minimise the potential sources of bias that we have identified in our study.

## Data Availability

The dataset generated and analysed during the current study is not publicly available because it could be possible to identify individuals from the dataset despite the dataset being anonymised.

## Abbreviations

ART: Antiretroviral therapy
CI: Confidence interval
DM: Diabetes mellitus
FBG: Fasting blood glucose
HIV: Human immunodeficiency virus
RBG: Random blood glucose
SQL: Structured query language
TB: Tuberculosis
